# Work-family conflicts and sickness absence due to mental disorders among female municipal employees – a register-linked study comparing health and social care employees to employees in other sectors

**DOI:** 10.5271/sjweh.4191

**Published:** 2024-12-01

**Authors:** Jaakko Harkko, Aino Salonsalmi, Noora A Heinonen, Tea Lallukka, Anne Kouvonen

**Affiliations:** * Shared first authorship; 1 Faculty of Social Sciences, University of Helsinki, Helsinki, Finland; 2 Department of Public Health, University of Helsinki, Helsinki, Finland; 3 Centre for Public Health, Queen’s University Belfast, Belfast, Northern Ireland

**Keywords:** mental health, sick leave, work-family interface

## Abstract

**Objectives:**

This study aimed to examine (i) if work-to-family conflicts (WTFC) and family-to-work conflicts (FTWC) are associated with sickness absence due to mental disorders and (ii) whether these associations are different among health and social care (HSC) employees compared to other municipal employee sectors.

**Methods:**

The Helsinki Health Study survey data collected in 2017 among 19–39-year-old female municipal employees (N=2557) were prospectively linked to administrative Social Insurance Institution of Finland register data on long-term sickness absence due to mental disorders (SA-MD) covering a follow-up of up to five years. The associations of WTFC and FTWC and SA-MD were analyzed using Cox regression models stratified by employment sector (HSC, education, other), adjusting for sociodemographic and health-related covariates.

**Results:**

Of HSC employees, 16% had SA-MD during the follow-up, which surpassed the figures for employees in education (12%) and other (11%) sectors. In the HSC sector, the youngest employees had the highest prevalence of SA-MD and, among HSC employees, prior SA-MD was the most common. In Kaplan–Meier curves, the steepest increase in SA-MD was observed for HSC employees. WTFC [hazard ratio (HR) 1.84, 95% confidence interval (CI) 1.39–2.45] and FTWC (HR 1.78, 95% CI 1.32–2.40) were associated with SA-MD among HSC employees. The associations were rather similar for employees in education and other sectors. Adjusting for work-related factors and health history somewhat attenuated the associations.

**Conclusions:**

Better possibilities to combine work and family life might aid in preventing SA-MD in all employment sectors.

Mental disorders are amongst the key reasons behind sickness absence in both Finland (1) and other OECD countries (2). In Finland, there was a steep increase in sickness absence due to mental disorders (SA-MD) between 2016 and 2019, particularly among young and early midlife women (3). Previous studies suggest that health and social care (HSC) employees have an increased risk of SA-MD compared to employees not working in human service (4–7). SA-MD is a key indicator of later permanent work disability (8), thus identifying risk factors behind it is vital for the HSC sector, which struggles with severe employee shortages globally (9). In addition, there are implications for patients and clients since poor mental health among HSC employees has been associated with medical errors (10) and deteriorated patient safety (11).

A reason behind excess SA-MD among HSC employees might be the pressures to fulfil the demands of both work and family life. These pressures are described as work–family conflicts (WFC) (12, 13), which can be seen as an inter-role conflict that occurs when the role pressures from work and family roles are incompatible (12, 13). The reasoning behind this is that individuals have to make use of their limited resources when performing multiple roles, which then results in inter-role conflicts. These conflicts are called work-to-family conflicts (WTFC) when experiences and commitments at work interfere with family life and family-to-work conflicts (FTWC) when family responsibilities (eg, providing informal care and taking care of children) affect work performance (12, 13). HSC employees might be especially vulnerable to the pressures of combining work and family since they typically engage in work requiring social and emotional contributions (14), and are exposed to violence (15), irregular working hours and shift work, predisposing them to WFC (16). In addition, the sector is predominantly occupied by women, who have more SA-MD (3) and report more WFC compared to men (17). On the other hand, the risk of SA-MD has been found to be even higher among men working in this sector (6). Studies on occupational and employee sector differences in WFC are largely lacking. A Norwegian study found that employees who worked in advertising or as church ministers or lawyers reported the most WFC, whereas bus drivers, teachers, and nurses reported the least WFC (18).

Previous studies have reported an association between WFC and sickness absence (17, 19–21), but to our best knowledge only two previous studies have examined diagnosis-specific sickness absence. A Swedish study of 19–47-year-old twins found that, among women, WTFC and FTWC were associated with sickness absence due to stress-related diagnoses and other mental diagnoses (22). A Finnish study of 40–55-year-old female municipal employees found associations between both WTFC and FTWC and SA-MD (23). In addition, a French study examined the combined effect of dependants at home and work stress among 46–55-year-old employees of the national gas and electricity company and reported that employees exposed to high levels of family and work demands had a higher risk for sickness absence due to non-psychotic mental disorders (24). As far as we are aware, prior studies have not looked at different employee sectors and only one that examined Norwegian nurses specifically focused on HSC employees’ WFC and sickness absence (25). WTFC was associated with all-cause sickness absence, whereas FTWC was not (25). However, the response rate was low and sickness absence was self-reported.

Sickness absence is a measure of health, but other factors such as family type and domestic responsibilities (26) and working conditions (27) are also associated with it. Since sociodemographic factors, working conditions and health behaviors are associated with sickness absence and WFC (13, 28), they are likely to contribute to the associations between WFC and sickness absence. In a Swedish study, after including health- or family-related factors no association between WFC and sickness absence due to mental diagnoses remained among women (22), whereas in a French study the associations were reduced but remained after adjustment for sociodemographic, behavioral and health characteristics (24). In an earlier Finnish study, associations were still observed after adjusting for family-related factors, working conditions and self-rated health (23).

This study aimed to examine whether WTFC and FTWC are associated with SA-MD among young and early midlife female municipal employees. A further aim was to investigate if these associations are different among HSC employees compared to employees working in other municipal sectors. Additionally, the study aimed to examine if various family- and work-related factors and health history contribute to the associations.

## Methods

### Study cohort

The Helsinki Health Study is a cohort study that explores the health and wellbeing of employees of the City of Helsinki (29). The City of Helsinki employs nearly 38 000 people annually in different occupations ranging from manual workers to routine non-manual staff, professionals and managers in various employment sectors (30). The largest employment sectors are HSC and education.

The target population consisted of 11 459 employees, 8801 of whom were women born in 1978 or later, and who had a job contract of ≥50% of regular work hours per week and lasting ≥4 months before the start of the data collection. The baseline survey was conducted in 2017 mainly via online or mailed questionnaire. In addition, telephone interviews with fewer questions than the original questionnaire were conducted among employees who did not otherwise respond. Of the participants, 43% worked in HSC, 35% in education and 23% in other employee sectors. The overall response rate was 51.5% (53% among women), with 5498 employees participating. Of the participants, 79% were women (N=4631), which corresponds to the gender distribution of the Finnish municipal sector. Overall, 82% of the participants consented to linking their questionnaire data with the employer’s and national registers. The telephone interviews lacked questions on WFC, thus only women who responded via online or mailed surveys were included in the study sample. Initially, the sample consisted of 3298 female employees working full- or part-time at the time of the survey who had consented to register linkage. Participants were excluded due to incomplete data (N=692), not having a family (N=222), and being on sickness absence during the survey (N=51). The exclusion criteria partially overlapped, and the final analytical sample was 2557 participants.

The non-response analyses showed that employees in higher socioeconomic positions and with less long-term sickness absence were somewhat more likely to participate, but overall the participants represent the target population fairly well (29). The response method (mail, online, telephone interview) was unlikely to have a strong impact on the factors associated with response and the impact of employment sector was negligible (29).

The Ethics Committee of the Faculty of Medicine at the University of Helsinki approved the study protocol, and permissions were granted from the City of Helsinki authorities. The register data holders granted access to link the register data with the survey data. Linking of participants’ survey responses to register data is based on their informed consent.

### Work–family conflicts

A questionnaire adapted from the National Study of Midlife Development in the US (31) was used to enquire about WTFC and FTWC. The items inquiring about WTFC were: ‘Your job reduces the amount of time you can spend with your family’, ‘Problems at work make you irritable at home’, ‘Your work involves a lot of travel away from home’, and ‘Your job takes so much energy you don’t feel up to doing things that need attention at home’. The item inquiring about travelling was omitted because travelling due to work was rare, and the data suggested that including the item reduced the reliability of the WTFC variable. A similar solution was made in previous studies focusing on an older cohort of the Helsinki Health Study (23, 32). The items about FTWC were: ‘Family matters reduce the time you can devote to your job’, ‘Family worries or problems distract you from your work’, ‘Family activities stop you from getting the amount of sleep you need to do your job well’, and ‘Family obligations reduce the time you need to relax or be by yourself’. Each question had four response alternatives: ‘not at all’, ‘to some degree’, ‘a lot’ and ‘I do not have a family’. As previously mentioned, individuals reporting that they had no family were excluded from the study. Of participants who reported not having children in other survey questions, approximately 11% chose the option ‘I do not have a family’. The individual items for WTFC and FTWC were summed to form composite variables, with higher scores indicating more conflicts. The score scores ranges were 3–9 and 4–12 for WTFC and FTWC, respectively. The Pearson correlation coefficient between continuous WTFC and FTWC variables was 0.246, indicating a weak positive correlation. The reliability of the variables was assessed using Cronbach’s alpha coefficients, which were 0.63 and 0.75 for WTFC and FTWC, respectively. The composite variables were divided into quartiles, and dummy variables were formed by classifying employees in the highest quartiles as having WFC. The cut-off point for both WTFC and FTWC was 7 points.

### Sickness absence due to mental disorders

Data on long-term SA-MD were received from the registers of the Social Insurance Institution of Finland. In Finland, all permanent residents aged 16–67 years are entitled to sickness allowance compensating for income loss because of work disability due to illness. The employer covers sickness absence of <12 calendar days. The Social Insurance Institution of Finland covers longer sickness absence lasting ≤300 calendar days, which requires a medical certificate with diagnosis-specific data. Diagnoses belonging to the class of mental and behavioral disorders (F00–F99) in the International Classification of Diseases (ICD-10) were defined as long-term SA-MD. Questionnaire data were prospectively linked with sickness absence data over five years from the time the respondent had responded to the survey. The linkage was done using national ID numbers assigned to Finnish citizens at birth and migrants upon receiving a residence permit. The follow-up ended at the first occurrence of long-term SA-MD during the follow-up or at the end of the five-year follow-up time. The number of days until the occurrence of SA-MD during the five-year follow-up was used as the outcome.

### Background variables

Information on employment sector was retrieved from the personnel register of the City of Helsinki. The employment sector was divided into three categories: HSC, education, and other. The most prevalent occupations in the HSC sector were clinical nurse, community health nurse, and care worker, and – in the education sector – daycare worker, kindergarten teacher, and primary school teacher. The ‘other’ sector was diverse, with the most frequent occupations being youth worker, tram driver, and project manager.

Regarding sociodemographic factors, participants’ ages were categorized into three categories: <30, 30–34, and 35–39 years. Marital status was divided into married or cohabiting versus single (including unmarried, divorced, or widowed individuals). The presence of children in the household was assessed as a dichotomous variable (yes/no). The highest education level of the employee was categorized into low (lower secondary school, vocational school, and matriculation examination), intermediate (bachelor’s degree and university of applied sciences degree) and high (master’s, licentiate and doctoral degrees).

Mental strenuousness of work was measured by a single-item question with four response alternatives and were recategorized as non-strenuous (rather or very light), intermediate (rather strenuous) and strenuous (very strenuous). Employment type was categorized as regular day job and other. The ‘other’ category included the options of regular night work; shift work without night work; shift work including night shift; day job, which includes night shifts; and other irregular working hours.

As for health-related factors, participants’ general health was assessed using the general health subscale of the RAND-36 (33). Employees in the lowest quartile were classified as having poor general health. The number of short-term sickness absence periods (<12 days) during one year preceding the baseline survey was derived from the employer’s personnel register and divided into low (0–1), intermediate (2, 3) and high (4–25). The thresholds were set to attain roughly equally sized groups and fulfil data protection requirements. Prior long-term SA-MD occurring before the survey return date was categorized as none, over two years ago, or within two years.

**Figure 1:**
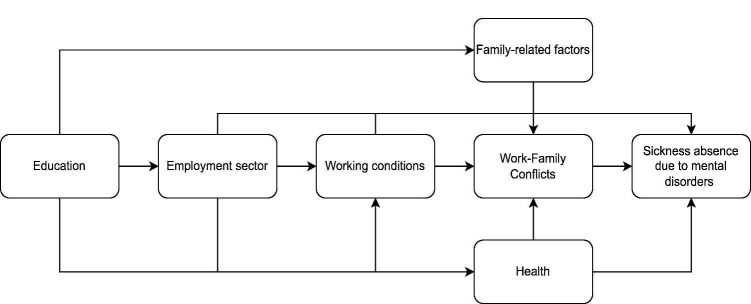
Directed acyclic graph of the assumed associations between employment sector, work–family conflicts, sickness absence due to mental disorders and covariates.

Assumed associations and pathways between WFC, SA-MD and background variables are shown with the means of directed acyclic graph in figure 1.

### Statistical analyses

Kaplan–Meier product limit estimates with survival graphs stratified by employment sector were constructed. Cox regression was used to analyze the associations between WFC and the first occurrence of SA-MD during the follow-up. The results are presented as hazard ratios (HR) and their 95% confidence intervals (CI). The base model (Model 1) was adjusted for age, with consecutive models additionally adjusted for marital status and children in the household; education; mental strenuousness of work and employment type; and general health, previous SA-MD and frequency of short sickness absence before baseline. There was no interaction between prior SA-MD and WFC. Consequently, the results are presented for the entire cohort instead of stratified analyses. The interactions between WFC and employment sector were tested and no interaction effects were found. A Schoenfeld test evaluated the proportional hazards assumption for all variables, and no assumption violations were revealed. The analyses were only conducted among women since the number of men was too small to allow for stratified analyses. The gender differences between the main study variables are presented in the supplementary material (www.sjweh.fi/article/4191) table S1.

As a sensitivity analysis, we replicated all analyses using data from participants with no prior SA-MD (N=2212). The robustness of the results was confirmed, with no significant deviations observed in the results (supplementary tables S2 and S3 and figure S1). All statistical analyses were performed using Stata, version 18 (StataCorp, College Station, TX, USA).

## Results

Table 1 presents the participants' background characteristics by employment sector. WTFC were more common in HSC and education compared to other sectors, whereas FTWC were most common in other sectors followed by the HSC sector. Of HSC employees, 16% had SA-MD during the follow-up, while this was 12% and 11% in education and other sectors, respectively.

**Table 1:** Characteristics of Helsinki Health Study participants (N = 2557) by employment sector. The prevalence of long-term sickness absence days due to mental disorders (SA-MD) (>11 calendar days) over a 5-year follow-up, N (%) by work–family conflicts and covariates stratified by employment sector.

		Health and social care			Education			Other	
		N (%)	SA-MD (%)		N (%)	SA-MD (%)		N (%)	SA-MD (%)
Total		1296 (100)	206 (15.9)		899 (100)	111 (12.3)		362 (100)	41 (11.3)
Months at risk, N (average)		71 016 (54.8)			49 982 (55.6)			20 254 (56.0)	
Work-to-family conflicts									
	Low	969 (76.7)	130 (13.4)		689 (76.6)	77 (11.2)		293 (80.9)	25 (8.5)
	High	302 (23.3)	76 (23.2)		210 (23.4)	34 (16.2)		69 (19.1)	16 (23.2)
Family-to-work conflicts									
	Low	994 (76.7)	139 (14.0)		720 (80.1)	77 (10.7)		266 (73.5)	25 (9.4)
	High	302 (23.3)	67 (22.2)		179 (19.9)	34 (19.0)		96 (26.5)	16 (16.7)
Age (years)									
	19–29	463 (35.7)	79 (17.1)		292 (32.5)	34 (11.6)		89 (24.6)	10 (11.2)
	30–34	418 (32.3)	70 (16.7)		299 (33.3)	39 (13.0)		128 (35.4)	11 (8.6)
	35–39	415 (32.0)	57 (13.7)		308 (34.3)	38 (12.3)		145 (40.1)	20 (13.8)
Marital status									
	Single	459 (35.4)	88 (19.2)		306 (34.0)	46 (15.0)		118 (32.6)	21 (17.8)
	Married or cohabiting	837 (64.6)	118 (14.1)		593 (66.0)	65 (11.0)		244 (67.4)	20 (8.2)
Children in the household									
	No	768 (59.3)	130 (16.9)		538 (59.8)	64 (11.9)		208 (57.5)	21 (10.1)
	Yes	528 (40.7)	76 (14.4)		361 (40.2)	47 (13.0)		154 (42.5)	20 (13.0)
Education									
	Low	343 (26.5)	68 (19.8)		312 (34.7)	44 (14.1)		119 (32.9)	18 (15.1)
	Intermediate	688 (53.1)	110 (16.0)		215 (23.9)	31 (14.4)		100 (27.6)	10 (10.0)
	High	265 (20.4)	28 (10.6)		372 (41.4)	36 (9.7)		143 (39.5)	13 (9.1)
Mental strenuousness									
	Non-strenous	205 (15.8)	14 (6.8)		133 (14.8)	15 (11.3)		146 (40.3)	10 (6.8)
	Intermediate	851 (65.7)	132 (15.5)		601 (66.9)	66 (11.0)		189 (52.2)	26 (13.8)
	Strenous	240 (18.5)	60 (25.0)		165 (18.4)	30 (18.2)		27 (7.5)	5 (18.5)
Employment type									
	Regular daytime	737 (56.9)	115 (15.6)		872 (97.0)	107 (12.3)		280 (77.3)	30 (10.7)
	Other	559 (43.1)	91 (16.3)		27 (3.0)	n<5		82 (22.7)	11 (13.4)
General health									
	Other	927 (71.5)	105 (11.3)		671 (74.6)	70 (10.4)		258 (71.3)	23 (8.9)
	Poor	369 (28.5)	101 (27.4)		228 (25.4)	41 (18.0)		104 (28.7)	18 (17.3)
Prior short sickness absence									
	Low	485 (37.4)	43 (8.9)		355 (39.5)	34 (9.6)		150 (41.4)	8 (5.3)
	Intermediate	403 (31.1)	62 (15.4)		252 (28.0)	29 (11.5)		116 (32.0)	16 (13.8)
	High	408 (31.5)	101 (24.8)		292 (32.5)	48 (16.4)		96 (26.5)	17 (17.7)
Prior sickness absence due to mental disorders									
	None	1099 (84.8)	130 (11.8)		795 (88.4)	80 (10.1)		318 (87.8)	27 (8.5)
	Over two years ago	131 (10.1)	48 (36.6)		74 (8.2)	22 (29.7)		27 (7.5)	7 (25.9)
	Within two years	66 (5.1)	28 (42.4)		30 (3.3)	9 (30.0)		17 (4.7)	7 (41.2)

Table 1 further shows that, among HSC employees, SA-MD was most common among the youngest two age groups, whereas in the other two sectors the prevalence was highest among older age groups. Employees reporting mentally strenuous work had the highest prevalence of SA-MD in all employment sectors. Health-related covariates were of particular importance with regards to SA-MD, with employees with poor general health, a high frequency of short sickness absence, and prior SA-MD having the highest prevalences of SA-MD.

**Figure 2:**
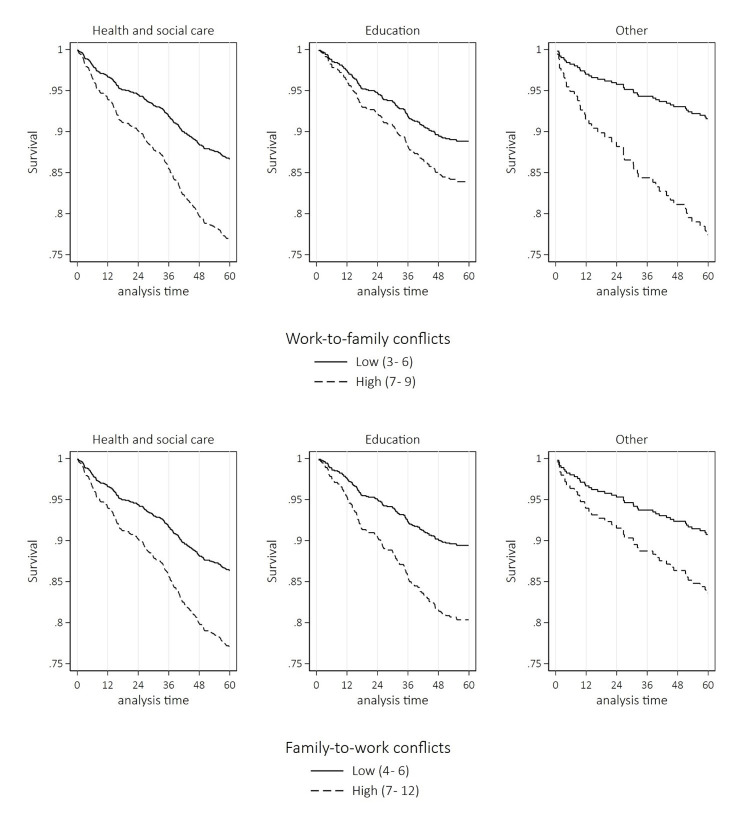
Cumulative probability of long-term sickness absence due to mental disorders among Helsinki Health Study participants by A) work-to-family and B) family-to-work conflicts stratified by employment sector [lysis time (months)].

Figure 2 shows Kaplan–Meier curves for time to long-term SA-MD by WFC stratified by employment sector. The graphs show a consistently different trend between employees experiencing WTFC and FTWC and those not experiencing them. The trends were generally similar between the employment sectors, but for both WTFC and FTWC the curves were the steepest among employees in the HSC sector.

In the HSC sector, high WTFC and FTWC were both associated with long-term SA-MD (table 2.). For participants with high WTFC, the age-adjusted HR was 1.84 (95% CI 1.39–2.45). Adjustment for family-related factors and education had only minimal effects, whereas adjustment for employment type and mental strenuousness of work attenuated the association while adjusting for health-related factors eliminated it. For FTWC, the HR started at 1.78 (1.32–2.40) in the age-adjusted model and decreased to 1.47 (1.09–1.98) in the model adjusted for health-related factors. Adjustment for family-related factors somewhat strengthened the association, whereas adjustment for education and employment type and mental strenuousness of work had no effect.

**Table 2:** The associations between work–family conflicts and sickness absence due to mental disorders over a 5-year follow-up stratified by employment sector among Helsinki Health Study participants. Hazard ratios (HR) and their 95% confidence intervals (CI) from Cox regression models.

Employment sector			Model 1 ^a^		Model 2 ^b^		Model 3 ^c^		Model 4 ^d^		Model 5 ^e^
			HR (95% CI)		HR (95% CI)		HR (95% CI)		HR (95% CI)		HR (95% CI)
**Health and social care**											
	Work-to-family conflicts										
		Low	1.00		1.00		1.00		1.00		1.00
		High	1.84 (1.39–2.45)		1.88 (1.41–2.49)		1.75 (1.31–2.34)		1.55 (1.13–2.11)		1.28 (0.95–1.73)
	Family-to-work conflicts										
		Low	1.00		1.00		1.00		1.00		1.00
		High	1.78 (1.32–2.40)		2.08 (1.50–2.90)		1.75 (1.30–2.36)		1.78 (1.32–2.40)		1.47 (1.09–1.98)
**Education**											
	Work-to-family conflicts										
		Low	1.00		1.00		1.00		1.00		1.00
		High	1.48 (0.99–2.22)		1.54 (1.03–2.31)		1.52 (1.02–2.29)		1.29 (0.83–2.00)		1.26 (0.83–1.90)
	Family-to-work conflicts										
		Low	1.00		1.00		1.00		1.00		1.00
		High	1.96 (1.29–2.96)		2.09 (1.33–3.29)		1.98 (1.31–3.01)		1.87 (1.23–2.85)		1.78 (1.18–2.71)
**Other**											
	Work-to-family conflicts										
		Low	1.00		1.00		1.00		1.00		1.00
		High	2.90 (1.54–5.44)		3.26 (1.73–6.15)		2.94 (1.56–5.53)		2.50 (1.30–4.82)		2.47 (1.28–4.74)
	Family-to-work conflicts										
		Low	1.00		1.00		1.00		1.00		1.00
		High	1.84 (0.96–3.53)		1.72 (0.87–3.40)		2.00 (1.04–3.85)		1.94 (1.01–3.71)		1.47–0.76 (2.82)

^a^ Adjusted for age. ^b^ Adjusted for Model 1 + Marital status, children in the household. ^c^ Adjusted for Model 1 + Education. ^d^ Adjusted for Model 1 + Employment type, mental strenuousness of work. ^e^ Adjusted for Model 1 + General health, prior sickness absence due to mental disorders, short sickness absence periods before baseline.

In the education sector, high WTFC was associated with SA-MD when adjusting for age and family-related factors (1.54, 1.03–2.31) as well as age and education (1.52, 1.02–2.29) (table 2.). Among employees with high FTWC, the age-adjusted HR was 1.96 (1.29–2.96), and adjusting for family-related factors somewhat strengthened the association while adjusting for health-related factors minimally attenuated it.

In the ‘other’ sector, the age-adjusted HR for those with high WTFC was 2.90 (1.54–5.44), this decreased to 2.50 (1.30–4.82) in the model adjusted for employment type and mental strenuousness of work and to 2.47 (1.28–4.74) in the model adjusted for health-related factors. Amongst employees with high FTWC, the models adjusted for age and education (HR 2.00, 95% CI 1.04–3.85) and age, employment type and mental strenuousness of work (HR 1.94, 95% CI 1.01–3.71) were statistically significant.

## Discussion

Both WTFC and FTWC were associated with SA-MD among female HSC employees. Adjusting for mental strenuousness of work and work type somewhat attenuated the association between WTFC and SA-MD and, for health history, the associations between both types of WFC and SA-MD. The associations were largely similar between HSC employees and the other employment sectors, but the overall prevalence of SA-MD was larger in the HSC sector.

In line with previous studies, both WTFC and FTWC were associated with SA-MD among women (22–24). In light of the theory of WFC as an inter-role conflict, it was not surprising that associations were found concerning both types of WFC since the theory suggests that WFC occurs when individuals have limited resources to draw upon to fulfil the demands of both roles. The different directions of WFC are, however, not interchangeable since they portray different dimensions of WFC and have partly different antecedents (13). Supporting this, there was only weak positive correlation between WTFC and FTWC in our study.

Together with previous studies, our study highlights the importance of WFC to SA-MD among female employees. Two of the three previous studies (22–24) were conducted in Nordic countries with a long history of dual-carer couples and family and work policies encouraging both parents to participate in work life (34). For example, in Finland all children under school-age have a subjective right to early childhood education and care. The studies indicate that improvements in policies regarding combining work and family are still needed. Future studies are needed to find identify if flexibilities in work time, family leave, and leaves to take care of older family members could aid in reducing SA-MD.

The novel finding of our study was that the associations were largely similar among HSC employees compared to those in education and other sectors. However, the Kaplan–Meier curves were the steepest among HSC employees for both types of WFC, reflecting that HSC employees had more SA-MD and WFC compared to other employees. The finding of extra SA-MD among HSC employees is in line with previous Nordic studies reporting (i) an increased risk of all-cause long-term sickness absence among HSC employees (35, 36), (ii) female health professionals and social workers having more SA-MD compared to employees not working in human service occupations (6), and (iii) female health professionals having more sickness absence due to depression compared to teachers (4). In previous studies, educational level seemed to play a role since medical doctors and psychologists did not have an elevated risk (6), and in another study upper non-manual and lower non-manual HSC employees in particular had a higher annual risk of SA-MD (5). In our study, there was a gradient of SA-MD among HSC employees by education, thus our results conform to the abovementioned ones. In our study, in the HSC sector – compared to the other sectors – the youngest employees had the highest incidence of SA-MD and prior SA-MD was more common among employees in HSC compared to the other sectors. The results thus suggest that among HSC employees, mental health problems start early on in their careers or alternatively individuals vulnerable to mental health problems are selected into HSC occupations. All in all, preventive measures are needed already starting from the training stage to improve work ability and tackle the employee shortage in the HSC sector. Previous studies on occupational and employee sector differences in WFC are largely lacking. Our results contrast with a Norwegian study that did not find an elevated level of WFC among nurses (18). An earlier Finnish study reported that municipal employees with children had lower opportunities to influence their work timing and the length of their workday than government and private sector employees or entrepreneurs (37). Further studies are needed to examine possible differences and the reasons behind them between occupations, employers, and employment sectors.

Adjustments were made for various family-, work-, and health-related factors. Unexpectedly, marital status and underage children did not attenuate the associations. Unfortunately, we had no data on the age of the children or single-parenting, which might have contributed to the results (38). Previous literature suggests that having young children is more strongly associated with WFC than having older children (39). However, taking care of sick or older family members can also be a source of WFC (40). Not being married is associated with mental health problems (41), and mental health problems are associated with not having children (42), which might bias the results regarding family-related factors. Education had essentially no effect, although HSC employees as well as employees in other sectors with the lowest education had the highest prevalence of SA-MD. Mental strenuousness of work and work type partially explained the associations between WTFC and SA-MD in all studied employment sectors. The finding is in line with previous evidence suggesting that working conditions are important antecedents of WTFC and non-work factors of FTWC (13). Health-related covariates attenuated and partially abolished the associations between WFC and SA-MD. The results thus imply that paying attention to mental strenuousness of work, poor general health, frequent short sickness absence and prior SA-MD might aid in preventing future SA-MD in all employment sectors. Adjustment by health history should, however, be interpreted with care since there is a risk of over-adjustment.

The strengths of the study include the large dataset, the opportunity to include several covariates, the prospective design and the reliable register-based diagnosis-specific data on sickness absence. The follow-up time was rather long, but the Kaplan–Meier curves showed that the associations between WFC and SA-MD did not depend on time. The baseline survey had a reasonably good response rate, but not all participants consented to linking their questionnaire data with register data and there were missing values due to other reasons. It might be that employees with strenuous family situations or mental health problems responded less often than others, which might dilute the associations. The Cronbach’s alpha of the WTFC was 0.63. A modest alpha suggests that the answers to the questions on WTFC were not completely consistent, which could reduce the reliability of the results. The study only included female municipal employees, thus the results cannot be generalized to all employees. The number of men in the dataset was too small to allow for stratified analyses by gender. Supplementary table S1 shows that the main study variables were differently distributed between genders, and thus pooling women and men was not feasible. Women in general had more SA-MD than men, but in the HSC sector the prevalence of SA-MD was equal among genders. Men reported more FTWC and women more WTFC.

### Concluding remarks

This study found that WTFC and FTWC were both associated with SA-MD among young and early midlife female municipal employees. The associations were largely similar between HSC employees and the other employment sectors, but the overall prevalence of SA-MD was larger in the HSC sector. SA-MD might be most effectively addressed through a dual approach implementing both general and employee sector–specific measures. Supporting employees with mentally strenuous work and paying attention to employees with indicators of poor health might aid in preventing SA-MD associated with WFC. In the HSC sector, paying attention to mental health problems already from the training stage onward might be useful.

## Supplementary material

Supplementary material
