# Supplementary material for: Work-family conflicts and sickness absence due to mental disorders among female municipal employees – a register-linked study comparing health and social care employees to employees in other sectors
Source: Scand J Work Environ Health. 2024 Nov 28;50(8):631–40. doi: 10.5271/sjweh.4191 (PMC11618849; doi:10.5271/sjweh.4191)
Supplement: Supplementary material [file SJWEH-50-631-S001.pdf]

Work-family conflicts and sickness absence due to mental disorders among female municipal employees – a register-linked study comparing health and social care employees to employees in other sectors[1]

by Jaakko Harkko, PhD, Aino Salonsalmi, MD, PhD,[2] Noora A Heinonen MHSc, Tea Lallukka, PhD, Anne Kouvonen, PhD

1. Supplementary material
2. Correspondence to: Dr Aino Salonsalmi, Department of Public Health University of Helsinki, PO Box 20 (Tukholmankatu 8 B), 00014 University of Helsinki, Finland. [E-mail: aino.salonsalmi@helsinki.fi]

Supplementary table S1. The characteristics of the Helsinki Health Study variables among women and men stratified by employment sector.

	**Health and social care**		**Education**		**Other**	
**Women**						
Total (n)	1296		899		362	
n with SA-MD (%)	206 (15.9)		111 (12.3)		41 (11.3)	
	n (%)	SA-MD	n (%)	SA-MD	n (%)	SA-MD
Work-to-family conflicts						
Low	969 (74.8)	130 (13.4)	689 (76.6)	77 (11.2)	293 (80.9)	25 (8.5)
High	327 (25.2)	76 (23.3)	210 (23.4)	34 (16.2)	69 (19.1)	16 (23.2)
Family-to-work conflicts						
Low	994 (76.7)	139 (14.0)	720 (80.1)	77 (10.7)	266 (73.5)	25 (9.4)
High	302 (23.3)	67 (22.2)	179 (19.9)	34 (19.0)	96 (26.5)	16 (16.7)
**Men**						
Total (n)	228		154		344	
n with SA-MD (%)	35 (15.4)		10 (6.5)		22 (6.4)	
	n (%)	SA-MD	n (%)	SA-MD	n (%)	SA-MD
Work-to-family conflicts						
Low	187 (82.0)	24 (12.8)	125 (81.2)	6 (4.8)	299 (86.9)	18 (6.0)
High	41 (18.0)	11 (26.8)	29 (18.8)	<5	45 (13.1)	<5
Family-to-work conflicts						
Low	163 (71.5)	19 (11.7)	110 (71.4)	9 (8.2)	258 (75.0)	16 (6.2)
High	65 (28.5)	16 (24.6)	44 (28.6)	<5	86 (25.0)	6 (7.0)

Supplementary table S2. Characteristics of the Helsinki Health Study participants with no prior long-term sickness absence due to mental disorders (n = 2212) by employment sector. The prevalence of sickness absence days due to mental disorders (> 11 calendar days) over a 5-year follow-up, n (%) by work–family conflicts and covariates stratified by employment sector.

	**Health and social care**		**Education**		**Other**	
**Total**	All	SA-MD	All	SA-MD	All	SA-MD
	1099 (100.0)	130 (11.8)	795 (100.0)	80 (10.1)	318 (100.0)	27 (8.5)
**Months at risk**, N (average)	61853 (56.3)		44981 (56.6)		18182 (57.2)	
	n (%)	SA-MD (%)	n (%)	SA-MD (%)	n (%)	SA-MD (%)
**Work-family-conflicts**						
Work-to-family conflicts						
Low	849 (77.3)	87 (10.2)	619 (77.9)	57 (9.2)	260 (81.8)	16 (6.2)
High	250 (22.7)	43 (17.2)	176 (22.1)	23 (13.1)	58 (18.2)	11 (19.0)
Family-to-work conflicts						
Low	868 (79.0)	94 (10.8)	651 (81.9)	61 (9.4)	240 (75.5)	17 (7.1)
High	231 (21.0)	36 (15.6)	144 (18.1)	19 (13.2)	78 (24.5)	10 (12.8)
**Covariates**						
Age						
19–29	423 (38.5)	58 (13.7)	273 (34.3)	27 (9.9)	82 (25.8)	9 (11.0)
30–34	348 (31.7)	42 (12.1)	266 (33.5)	29 (10.9)	119 (37.4)	7 (5.9)
35–39	328 (29.8)	30 (9.1)	256 (32.3)	24 (9.4)	117 (36.8)	11 (9.4)
Marital status						
Single	382 (34.8)	57 (14.9)	266 (33.5)	30 (11.3)	96 (30.2)	11 (11.5)
Married or cohabiting	717 (65.2)	73 (10.2)	529 (66.5)	50 (9.5)	222 (69.8)	16 (7.2)
Children in the household						
No	667 (60.7)	87 (13.0)	489 (61.5)	49 (10.0)	185 (58.2)	15 (8.1)
Yes	432 (39.3)	43 (10.0)	306 (38.5)	31 (10.1)	133 (41.8)	12 (9.0)
Education						
Low	270 (24.6)	43 (15.9)	263 (33.1)	26 (9.9)	102 (32.1)	11 (10.8)
Intermediate	600 (54.6)	71 (11.8)	196 (24.7)	25 (12.8)	89 (28.0)	6 (6.7)
High	229 (20.8)	16 (7.0)	336 (42.3)	29 (8.6)	127 (39.9)	10 (7.9)
Mental strenousness						
Non–strenous	182 (16.6)	9 (4.9)	123 (15.5)	11 (8.9)	132 (41.5)	5 (3.8)
Intermediate	724 (65.9)	86 (11.9)	537 (67.5)	48 (8.9)	160 (50.3)	17 (10.6)
Strenous	193 (17.6)	35 (18.1)	135 (17.0)	21 (15.6)	26 (8.2)	5 (19.2)
Employment type						
Regular daytime	631 (57.4)	73 (11.6)	770 (96.9)	77 (10.0)	247 (77.7)	21 (8.5)
Other	468 (42.6)	57 (12.2)	25 (3.1)	n<5	71 (22.3)	6 (8.5)
General health						

Other	823 (74.9)	69 (8.4)	612 (77.0)	53 (8.7)	232 (73.0)	15 (6.5)
Poor	276 (25.1)	61 (22.1)	183 (23.0)	27 (14.8)	86 (27.0)	12 (14.0)
Prior short sickness absence						
Low	438 (39.9)	29 (6.6)	327 (41.1)	26 (8.0)	137 (43.1)	n<5
Intermediate	353 (32.1)	43 (12.2)	228 (28.7)	25 (11.0)	101 (31.8)	10 (9.9)
High	308 (28.0)	58 (18.8)	240 (30.2)	29 (12.1)	80 (25.2)	13 (16.3)

Supplemental Figure 1. Cumulative probability of long-term sickness absence due to mental disorders among Helsinki Health Study participants with no prior long-term sickness absence due to mental disorders by a) work-to-family and b) family-to-work conflicts stratified by employment sector.

a)

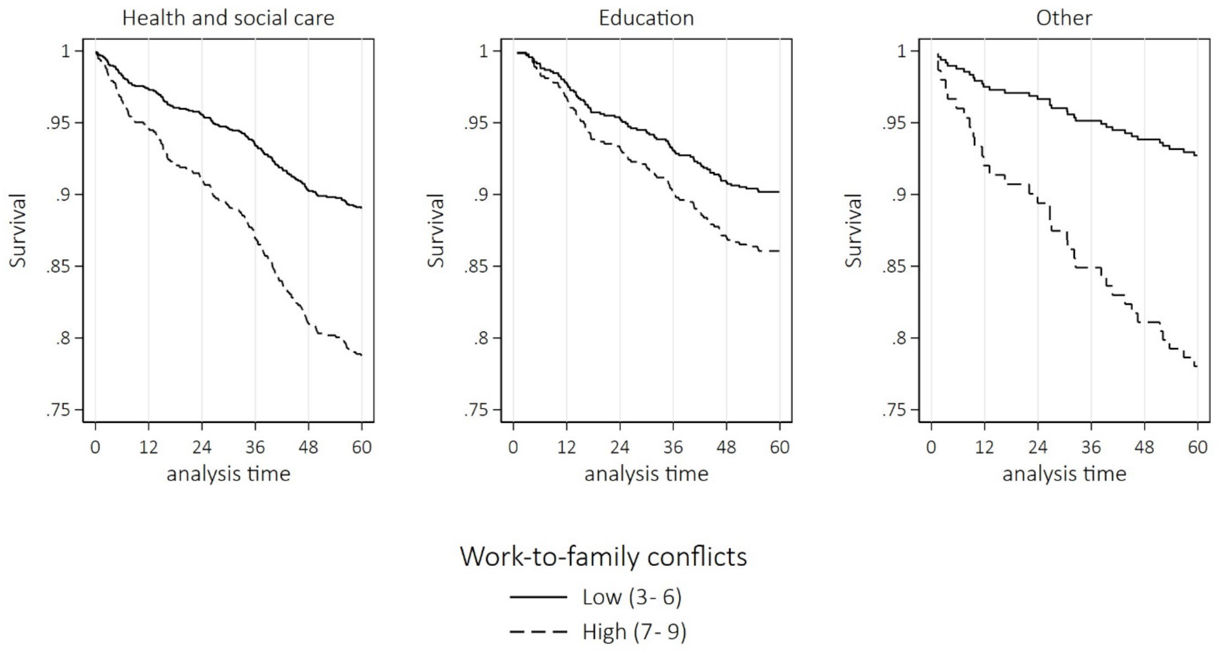

b)

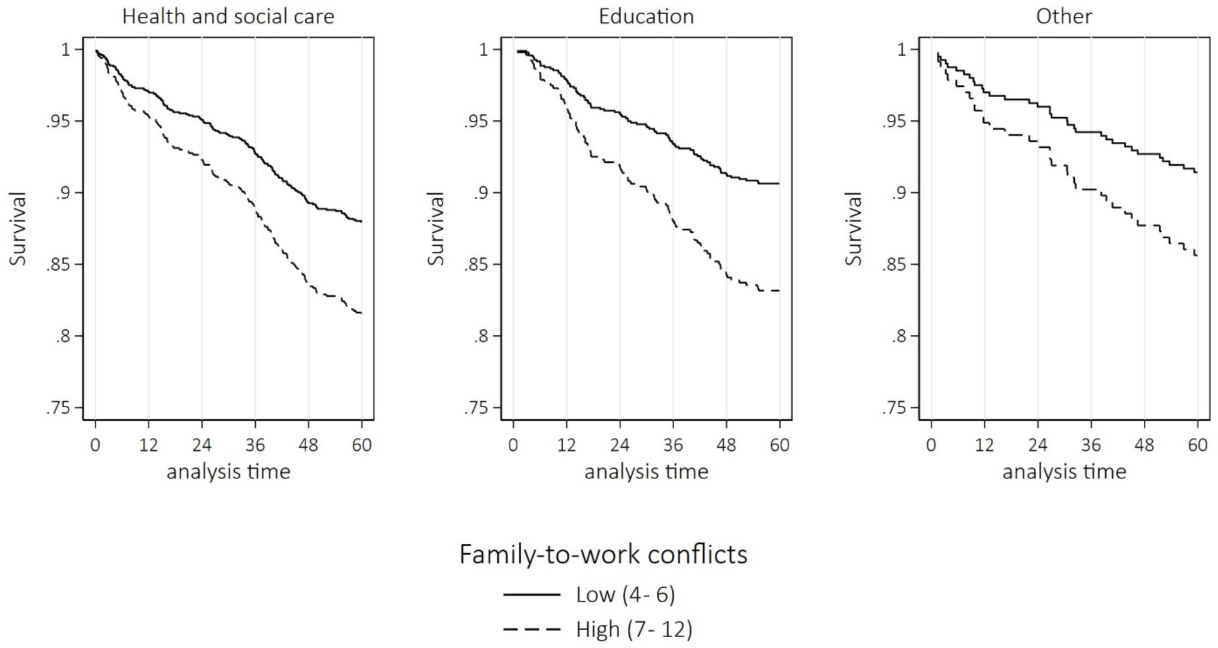

analysis time (months)

Supplementary Table 3. The associations between work–family conflicts and sickness absence due to mental disorders over a 5-year follow-up stratified by employment sector among Helsinki Health Study participants with no prior long-term sickness absence due to mental disorders (n=2212). Hazard ratios and their 95% confidence intervals from Cox regression models.

Employment sector	Model 1	Model 2	Model 3	Model 4	Model 5
**Health and social care**					
Work-to-family conflicts					
Low	1.00	1.00	1.00	1.00	1.00
High	1.72 (1.19-2.48)	1.77 (1.22-2.55)	1.60 (1.10-2.32)	1.46 (0.98-2.17)	1.35 (0.93-1.96)
Family-to-work conflicts					
Low	1.00	1.00	1.00	1.00	1.00
High	1.65 (1.11-2.44)	1.94 (1.26-3.00)	1.62 (1.10-2.40)	1.65 (1.11-2.44)	1.47 (0.99-2.17)
**Education**					
Work-to-family conflicts					
Low	1.00	1.00	1.00	1.00	1.00
High	1.45 (0.89-2.35)	1.48 (0.91-2.40)	1.46 (0.90-2.38)	1.22 (0.72-2.08)	1.34 (0.82-2.19)
Family-to-work conflicts					
Low	1.00	1.00	1.00	1.00	1.00
High	1.50 (0.88-2.54)	1.56 (0.89-2.75)	1.53 (0.90-2.59)	1.44 (0.85-2.45)	1.48 (0.87-2.52)
**Other**					
Work-to-family conflicts					
Low	1.00	1.00	1.00	1.00	1.00
High	3.36 (1.56-7.27)	3.61 (1.66-7.86)	3.37 (1.56-7.27)	2.76 (1.24-6.13)	2.65 (1.20-5.84)
Family-to-work conflicts					
Low	1.00	1.00	1.00	1.00	1.00
High	2.09 (0.92-4.75)	2.05 (0.87-4.85)	2.16 (0.95-4.89)	2.17 (0.95-4.93)	1.96 (0.88-4.36)

M1: Age

M2: M1 + Marital status, children in the household

M3: M1 + Education

M4: M1 + Employment type, mental strenuousness of work

M5: M1 + General health, prior sickness absence due to mental disorders, short sickness absence periods before baseline
